# Chronic Inflammation in Non-Alcoholic Steatohepatitis: Molecular Mechanisms and Therapeutic Strategies

**DOI:** 10.3389/fendo.2020.597648

**Published:** 2020-12-14

**Authors:** Carmelo Luci, Manon Bourinet, Pierre S. Leclère, Rodolphe Anty, Philippe Gual

**Affiliations:** ^1^ Université Côte d’Azur, INSERM, C3M, Nice, France; ^2^ Université Côte d’Azur, CHU, INSERM, C3M, Nice, France

**Keywords:** NAFLD, NASH, inflammation, liver injury, macrophages, ILCs, hepatocytes, therapy

## Abstract

Non-Alcoholic Steatohepatitis (NASH) is the progressive form of Non-Alcoholic Fatty Liver Disease (NAFLD), the main cause of chronic liver complications. The development of NASH is the consequence of aberrant activation of hepatic conventional immune, parenchymal, and endothelial cells in response to inflammatory mediators from the liver, adipose tissue, and gut. Hepatocytes, Kupffer cells and liver sinusoidal endothelial cells contribute to the significant accumulation of bone-marrow derived-macrophages and neutrophils in the liver, a hallmark of NASH. The aberrant activation of these immune cells elicits harmful inflammation and liver injury, leading to NASH progression. In this review, we highlight the processes triggering the recruitment and/or activation of hepatic innate immune cells, with a focus on macrophages, neutrophils, and innate lymphoid cells as well as the contribution of hepatocytes and endothelial cells in driving liver inflammation/fibrosis. On-going studies and preliminary results from global and specific therapeutic strategies to manage this NASH-related inflammation will also be discussed.

## Introduction

**Figure 1:**
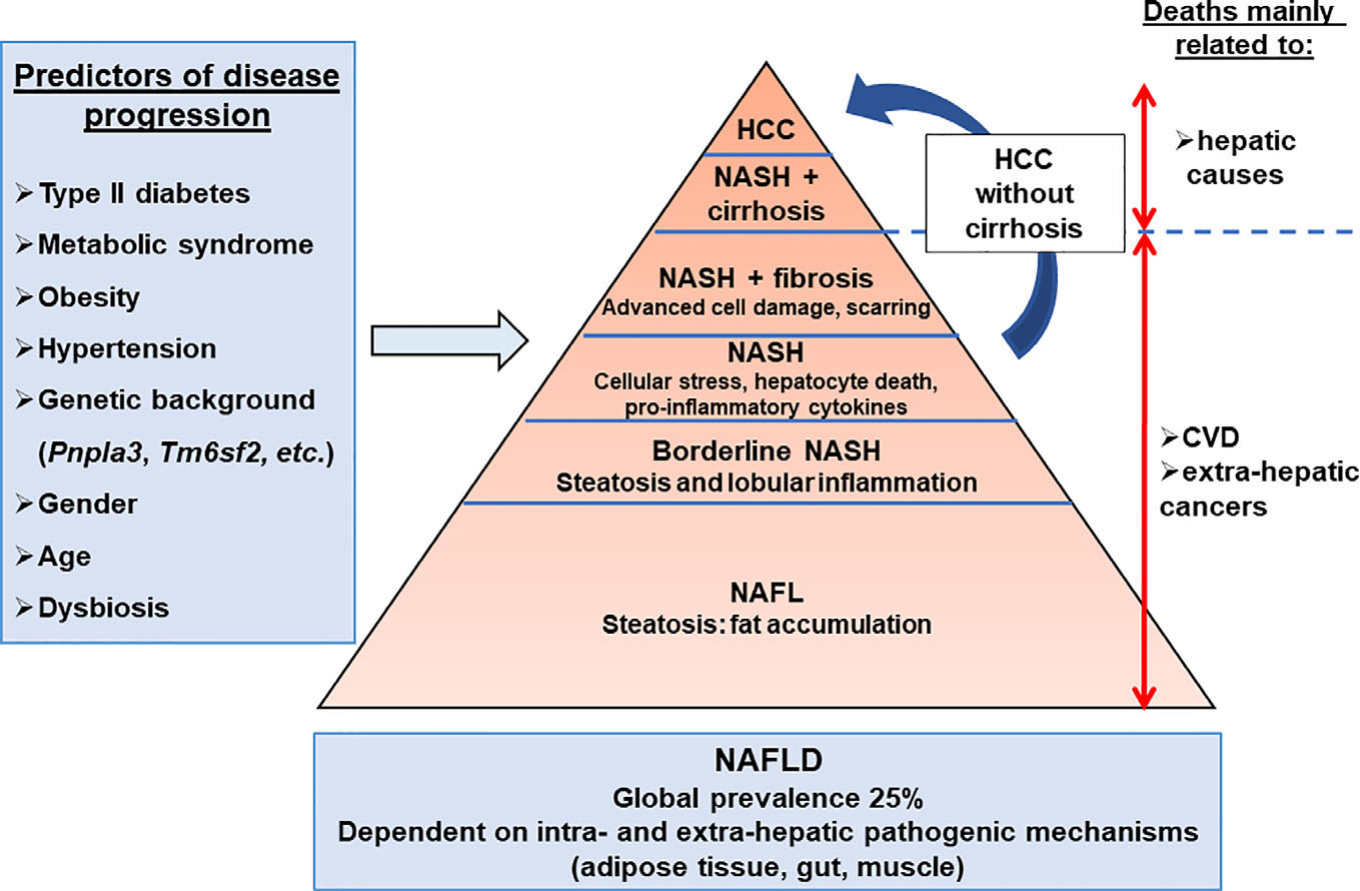
Hepatic complications associated with obesity. The NAFLD development and progression are influenced by environmental, genetic, and individual features. The main predictors of disease progression are the presence of type II diabetes, metabolic syndrome, hypertension, and dysbiosis. NAFLD progression is also influenced by genetic, epigenetic, gender, and age components. The dysregulation of extra-hepatic organ functions such as adipose tissue, gut, and muscle, as well as intra-hepatic events (inflammation, cell death, cellular stresses), have been reported in NAFLD. Cardiovascular disease (25–43%) is the primary cause of death in NAFLD patients, while liver-related disease (9–15%) is also substantial. NAFL, non-alcoholic fatty liver; NAFLD, non-alcoholic fatty liver diseases; NASH, non-alcoholic steatohepatitis.

Non Alcoholic Fatty Liver Diseases (NAFLDs), recently renamed Metabolic Associated Fatty Liver Diseases (MAFLDs) to better reflect the pathogenesis (1, 2), are the most common chronic liver diseases, with a worldwide prevalence of 25% (3, 4). NAFLDs covers the full spectrum of fatty liver disease from hepatic steatosis to Non-Alcoholic Steatohepatitis (NASH), fibrosis/cirrhosis and hepatocellular cancer. The overall prevalence of NAFLD is growing in parallel with the global epidemic of obesity (5). Weight gain, insulin resistance, type 2 diabetes mellitus, and hypertension are risk factors for NAFLD progression (6, 7). Reciprocally, NAFLD is a risk factor for many metabolic diseases, including cardiovascular disease (8) and type 2 diabetes (9). NAFLD occurrence appears to be higher in men (10, 11), while postmenopausal women display an increased risk of severe ﬁbrosis compared to men, which can probably be attributed to the loss of the protective effects of estrogen against ﬁbrogenesis (11). Age also impacts the NAFLD prevalence and liver disease stage (12). NASH, which is considered to be the progressive form of the hepatic disease, is the second principal indication for hepatic transplantation and the growing etiology for hepatocellular carcinoma (HCC) in patients being listed for the liver transplantation (16.2% of all liver transplantations) in USA (13, 14). Finally, specific pharmacological therapies are not yet approved for advanced NASH (**Figure 1**).

In addition to genetic and environmental factors, the interactions of the gut and adipose tissue with the liver enhance liver metabolic disorder (steatosis and insulin resistance), chronic inflammation and injury-mediated fibrosis (15). Adipose tissue plays an important role in the development of insulin resistance and NAFLDs. Trunk fat was found to be indicative of elevated ALT supporting the potential involvement of the metabolically active intra-abdominal fat in increased liver injury (16). Obesity is associated with an increase in adipose tissue lipolysis, and secretion of inflammatory and fibrotic mediators which can reach the liver. The accumulation of inflammatory/immune cells and the modification of the activities of these cells in the adipose tissue contributed to chronic low grade inflammation during obesity (17–24). This sustained inflammation mediates insulin-resistance and provides a contributing link between its development and NAFLD (25). The gut-liver axis is also a critical actor in the development of NAFLD. Gut dysbiosis is associated with the modulation of local immune systems and altered mucosal barrier integrity which, in turn, promotes the translocation of bacterial products (26). In concert with the local action, gut metabolites (decreased choline availability, increased trimethylamine, ethanol production, changes in short-chain fatty acids, secondary bile acids, and branched-chain ramified amino acids, etc.) and pathogen-associated molecular patterns (PAMPs) modulate the metabolic and immune responses within many organs including adipose tissue, muscle and liver (27, 28). In addition to endotoxemia (circulating LPS), changes in microbiota in blood are associated with hepatic fibrosis in obese patients and liver tissue contains substantial amounts of bacterial DNA correlating with the histological disease severity in NAFLD subjects (29, 30).

In liver with NAFLD, a large amount of innate and adaptive immune cells including resident and recruited monocytes, macrophages, neutrophils and ILCs but also parenchymal hepatocytes and liver sinusoidal endothelial cells (LSECs) are involved in the onset of chronic inflammation (24, 31–36). The hepatocytes, LSECs, resident macrophages (Kupffer cells) are able to sense excessive levels of metabolites, damage-associated molecular patterns (DAMPs), and PAMPs and in turn elicit inflammatory events associated with metabolic dysfunction (31, 36). Recruited monocyte-derived macrophages and neutrophils are also key players in the NAFLD onset and progression, and new roles for ILC subsets have recently been described for NAFLD/obesity (24, 31–33). The immunological functions of several conventional immune cells and liver cells (hepatocytes, LSECs) in the context of obesity and NAFLD will be described in this review, which will also provide insights into the potential approaches to target these responses as therapies against NASH.

## Liver Cells That Promote the Immunological Responses Associated With NASH

**Figure 2:**
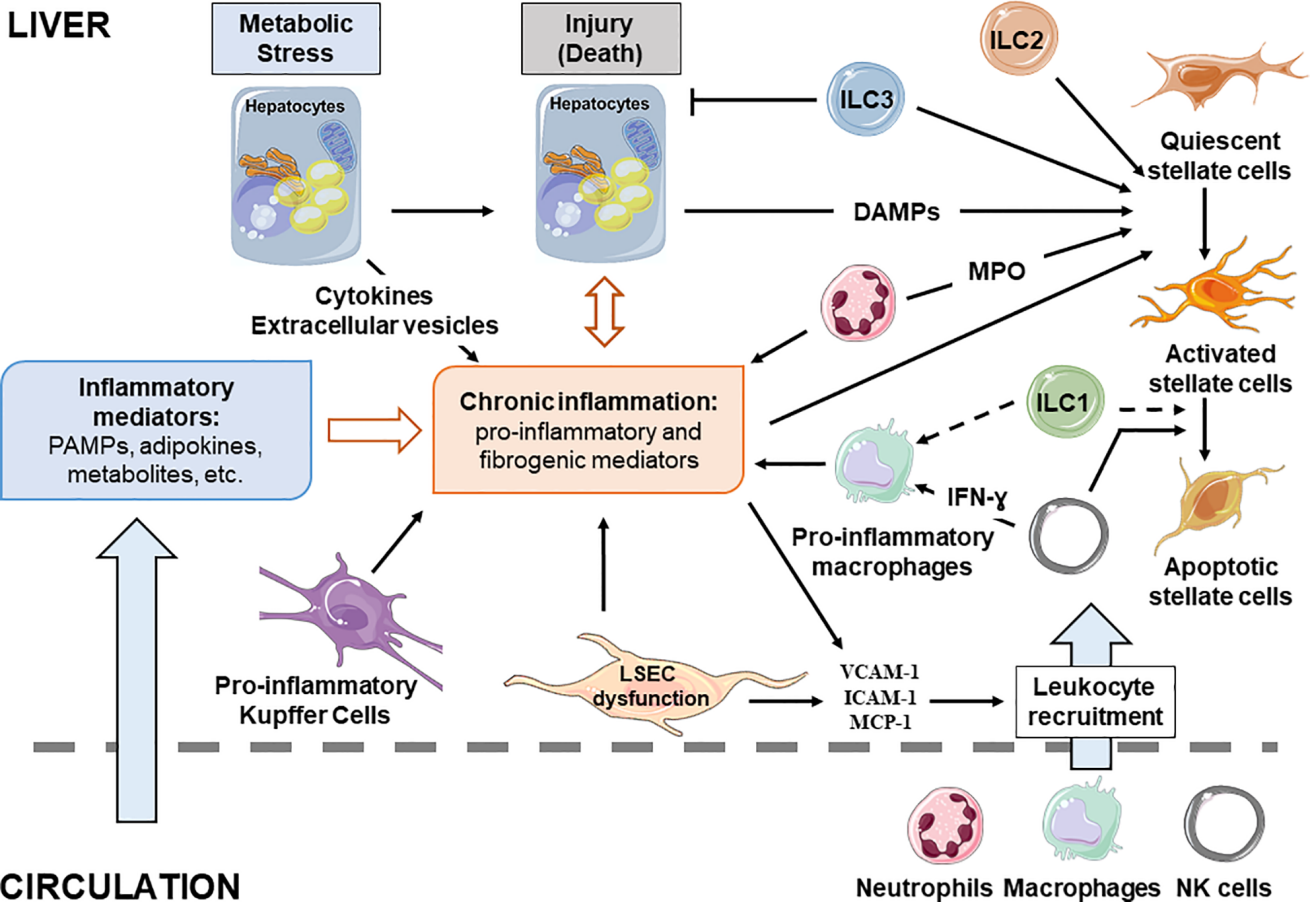
Cellular interplay during chronic liver diseases. Liver resident and recruited immune cells, stressed hepatocytes and liver sinusoidal endothelial cells contribute to development of the chronic inflammation associated with NAFLD. Inflammatory mediators reaching the liver are key contributors of disease progression as they influence hepatic cell functions. Full arrow, confirmed effect; dotted arrow, putative effect.

The main hepatic blood supply come from the gut via the portal vein (~80%). This blood supply is rich in toxins, food antigens and bacterial products from the environment. The hepatocytes, the most abundant liver cells (~70%), achieve the detoxifying and metabolic needs of the body. The remaining liver cells comprise the non-parenchymal cells (NPC), counting liver stellate cells (HSCs), sinusoidal endothelial cells (LSECs), and a variety of immune cells. The most abundant immune cells in the liver are resident macrophages, referred to as Kupffer cells (~20% among NPC). The liver also contains mucosal-associated invariant T (MAIT) cells, T cells, dendritic cells, natural killer cells (NK), neutrophils, iNKT, and ILCs. The inter-species variations in liver-resident immune cell populations need to be noted and taken into account for translational studies. For example, MAIT cells are one of the major liver populations of T cells in human (20–50% in human liver versus 0.4–0.6% in mouse liver) (37) and mouse liver contains less NK cells than human liver (5–10 versus 25–40% of total intrahepatic lymphocytes) (34). In addition to its detoxifying and metabolic roles, the liver is also a key immunological organ in the response to exogenous antigens, metabolites and pattern molecules (31, 38, 39). In the case of obesity, the liver sentinel cells such as monocyte-derived macrophages and resident Kupffer cells rapidly sense the local and persistent increase in pattern molecules, metabolites, and exogenous antigens. The liver then “transits” from an immune-tolerant state to an immune-active phenotype, with a shift in production of anti-inflammatory cytokines such as transforming growth factor-β (TGF β) and interleukin-10 (IL10) and to pro-inflammatory cytokines such as IL1, IL6, and tumor necrosis factor (TNF-α). In turn, the interplay between innate and adaptive immune cells and hepatocytes drives the chronic low grade inflammation in NASH liver (31). Without underestimating the important role played by the adaptive immune system [reviewed in (34, 35)], the immunological functions of hepatocytes and sentinel cells including LSECs, macrophages, ILCs, and neutrophils according to liver steatosis development and its progression to fibrotic-NASH, which will be debated in the following section (**Figure 2**, **Table 1**).

**Table 1:** Contribution of specific liver cells in the development of inflammation associated with NASH.

Type of cell	Roles in steatohepatitis	References
Hepatocytes	- sense PAMPs DAMPs, metabolite molecules (saturated FFA), and release inflammatory mediators (TNFα, IL1β) - hepatocyte death (lytic cell death > apoptosis) contribute to DAMPs induced inflammation - hepatocyte extracellular vesicles signals to immune cells	(31, 40, 41)
LSECs	- orchestrate release of proinflammatory mediators (cytokines chemokines such as MCP1, IL1/6, TNFα), (adhesion molecules such as VCAM1, ICAM) - enhance liver inflammation, injury, and fibrosis	(36, 42)
Resident and recruited macrophages	- sense PAMPs DAMPs and metabolites (saturated FFA), - contribute to the recruitment and activation of other hepatic immune cells via inflammatory chemokines and cytokines - specific subsets of liver macrophages enhance NAFLD progression	(32, 43)
NK cells/ILC1	- production of IFNγ and TNFα - regulate macrophage polarization towards a pro-inflammatory phenotype - display an anti-fibrogenic role	(24, 43, 44)
Neutrophils	- secretion of elastase, NETS - contribute to the onset of the early stage of NAFLD - promote hepatocyte injury	(45, 46)

### Hepatocytes Have an Important Role in Local Inflammation

The mechanisms involved in the steatosis-NASH transition are multifactorial and not completely elucidated. In hepatocytes, the accumulation of triglycerides in lipid droplets is a protective mechanism and prevents lipotoxicity by buffering the toxic free fatty acids (47). However, it is always a question of equilibrium and this protective mechanism can be overwhelmed. To illustrate that, the inhibition of the triglyceride synthesis through the targeting of diacylglycerol acyltransferase 2 (DGAT2) improves liver steatosis but exacerbates the liver injury and fibrosis in obese mice with steatohepatitis (48). In contrast, appropriate regulation of DGAT2 activity has been shown to have a protective effect against NASH (49). Altered lipid droplet remodeling or lipid mobilization can enhance hepatocyte lipotoxicity and drive NAFLD progression. In line with this, genetic variants in transmembrane 6 superfamily member 2 (TM6SF2) and patatin-like phospholipase domain-containing protein 3 (PNPLA3) genes, resulting in loss-of-function and decreased VLDL secretion and lipid droplet remodeling, respectively, were robustly linked to NAFLD development and its progression (50, 51). Furthermore, proteins involved in hepatic lipid homeostasis can be differentially expressed and mediate *de novo* NAFLD progression. For example, members of the CIDE family, which can regulate lipid droplet synthesis, hepatic lipid homeostasis and cell death, are differentially regulated according to NAFLD development and severity in mouse and human studies. The gradual increase in FSP27β/CIDEC2 expression with hepatic steatosis and then steatohepatitis could reflect the transition from the synthesis of protective lipid droplets to detrimental hepatocyte death. Indeed, the strong increase in FSP27β expression in NASH liver is more narrowly related to liver injury and its over-expression sensitized hepatocytes to cell death induced by TNFα and palmitic acid (52). The vulnerable fatty and stressed hepatocytes then release danger signals such as DAMPs, alarmins and apoptotic bodies. This activation of “sterile” inflammation contributes to the initiation of a vicious cycle, where inflammation enhances the death of hepatocyte and *vice versa*.

Different types of hepatocyte death have been associated with NASH-driven hepatic inflammation and NAFLD progression such as apoptosis, necrosis, necroptosis, pyroptosis, and ferroptosis (40, 41). Apoptotic hepatocytes can directly initiate inflammation such as the activation macrophage after engulfment of apoptotic bodies. Furthermore, hepatocyte apoptosis frequency and the levels of a circulating surrogate biomarker of hepatocyte apoptosis (caspase-generated keratine-18 fragments) increased with NASH and fibrosis (53, 54). Lytic forms of hepatocellular death, including necrosis, pyroptosis, necroptosis, and ferroptosis also cause strong inflammatory responses through the cellular components release such as DAMPs. These cellular components contribute to the recruitment and activation of inflammatory cells and hepatic stellate cells (41). In addition, the release of pro-inflammatory vesicles by stressed cells may also promote angiogenesis and activation of hepatic stellate cells. The release of a C-X-C motif chemokine ligand-10 (CXCL-10) and ceramide-enriched extracellular vesicles regulate liver trafficking and infiltration of monocytes and macrophages. Moreover, mitochondrial DNA and tumor necrosis factor-like apoptosis inducing ligand (TRAIL)-enriched extracellular vesicles promote macrophage activation (41). In addition, the senescence of hepatocytes is strongly correlated with the ﬁbrosis stage, type 2 diabetes and the clinical outcome (55). While hepatocyte senescence, mainly caused by HFD and aging, has a detrimental impact on hepatic steatosis (56), the senescent HSCs produce less extracellular matrix components and more matrix metalloproteinases, thereby alleviating fibrosis advancement (57). More studies are thus required in order to clarify the role of senescence in the different liver cells impacting the NAFLD progression.

Hepatocytes also sense pathogens and metabolic molecules via their membrane and cytoplasmic pattern recognition receptors (PRRs) [Toll-like receptor -2, -4, -5, and -9 (TLR); nucleotide-binding oligomerization domain -1 and -2 (NOD), cGMP-AMP synthase, etc.] (31). They are strongly involved in the regulation of the cell-autonomous innate immune responses leading to increased local inflammation and the development of liver complications (steatosis, insulin resistance and injury). Deficiency of TLR-2, TLR-4, or TLR-9 in hepatocytes resolved hepatic inflammation mediated by diet associated with decreased insulin resistance, oxidative stress and hepatic steatosis (58, 59). In contrast, the deficiency of TLR5 in hepatocytes (~90% of its hepatic expression) strongly impaired bacterial clearance (bacterial flagellin) by the liver and aggravated NAFLD development (from steatosis to liver injury and fibrosis) upon HFD or MCDD challenge (60). In addition to hepatocytes, other “non-conventional immune cells” such as LSECs are key actors in NAFLD development and hepatic inflammation.

### The Dysfunction of Sentinel LSECs Is Associated With NAFLD Progression From Hepatic Steatosis to Fibrosis

In physiological conditions, LSECs are gatekeepers of liver homeostasis. LSECs display anti-inﬂammatory and anti-ﬁbrogenic properties by preventing Kupffer cell and hepatic stellate cell activation and regulating hepatic lipid metabolism [reviewed in (36)]. Early in the course of NAFLD, LSEC capillarization leads to the loss of LSEC fenestrae and alters the transfer of chylomicron remnants to the hepatocytes required for the VLDL synthesis. As a compensatory mechanism, hepatocyte synthesis of cholesterol and triglycerides could be strongly increased. The synthesis and release of nitric oxide (NO) by LSECs also decreases with the dyslipidaemia and insulin resistance, altering the protective and local role of NO in the regulation of hepatocyte lipid content. Indeed, NO limits *de novo* lipogenesis and enhances the beta-oxidation of fatty acid in hepatocytes. During NASH, inﬂammation and gut microbiota-derived signals could increase the NF-kB pathway activation in LSECs, which coordinate the release of pro-inﬂammatory mediators including MCP1, IL1, IL6, TNFα, and the upregulation of adhesion molecules such as vascular cell adhesion molecule-1 (VCAM-1), intercellular adhesion molecule-1 (ICAM-1), and vascular adhesion protein-1 (VAP-1). This source of inflammatory mediators will amplify the local inflammation and liver injury by enhancing the hepatic recruitment and activation of leucocytes including macrophages and neutrophils. Altered LSECs also fail to maintain hepatic stellate cell quiescence and release ﬁbrogenic mediators, including Hedgehog signalling molecules, promoting liver ﬁbrosis. The decreased autophagic flux in LSECs, as evaluated by the incidence of autophagic vacuoles, has been recently associated with NASH in patients (42). In a mouse model of NAFLD (high-fat diet), the defect of autophagy in endothelial cells promotes liver inflammation (upregulation of inflammatory markers such as CCL2, CCL5, CD68, VCAM1) and injury (increased cleaved caspase-3 level) in addition to perisinusoidal fibrosis (42). As potential therapeutic approaches against NAFLD, activation of liver autophagy would thus be protective in hepatocytes, liver macrophages and sinusoidal endothelial cells, but detrimental in hepatic stellate cells (61, 62).

### Liver Macrophages are Important Drivers of Hepatic Inflammation

The role of the adipose tissue macrophages in the onset and progression of NAFLD by regulating, for example, local and systemic inflammation, insulin resistance, increased lipolysis and secreted pro-inflammatory and pro-fibrogenic adipokines has been well reported. The liver macrophages are also key actors in NAFLD pathogenesis (32, 33, 41). Liver-resident Kupffer cells are in close contact with LSECs in sinusoids and with hepatocytes and hepatic stellate cells in the parenchymal. At the early stage of NAFLD, the increased pro-inflammatory polarization of liver-resident Kupffer cells could contribute to hepatic steatosis and initiate inflammation and the recruitment of other immune cells into the liver. Indeed, the IL1β secretion by pro-inflammatory-Kupﬀer cells promotes accumulation of triglyceride in hepatocytes through the inhibition of peroxisome proliferator-activated receptor alpha (PPARα)-mediated fat oxidation (63). The decrease in anti-inflammatory macrophages in arginase-2 deﬁcient mice is also sufficient to promote the spontaneous development of liver steatosis mainly via the increase in *de novo* lipogenesis and inﬂammation dependent on iNOS (64). The depletion of Kupffer cells also attenuates hepatic steatosis and liver insulin resistance in rats fed high-sucrose or high-fat diets (65). In line with this, the anti-inflammatory Kupffer cells could mediate apoptotic effects towards their pro-inflammatory counterparts have been reported via the IL10 pathway. This could regulate the equilibrium between anti- and pro-inflammatory macrophages in the liver and prevent the early liver manifestation of metabolic syndrome (66). Bacterial products, toxic lipids, and hepatocyte-derived inflammatory mediators could amplify the pro-inflammatory polarization of liver macrophages with the increased chemokines secretion. In addition to resident macrophages, stressed hepatocytes, endothelium, and/or hepatic stellate cells contribute to this upregulation of liver chemokines leading to the recruitment of inflammatory cells (monocytes, neutrophils, lymphocytes) into the liver (67). The striking accumulation of immune cells is one hallmark characteristic of NASH and has been associated with ongoing hepatic inflammation and NAFLD progression (68, 69). With the NAFLD progression, it has been recently reported that the number of resident-Kupffer cells from embryonic progenitors is decreased causing by elevated cell death and are replaced by monocyte-derived Kupffer cells (70). This new pool of kupffer cells are more inflammatory and are important contributor of the impairment of liver responses during NASH (70). Important heterogeneity in liver macrophages from different origins thus exist and could be modify according to the NAFLD progression. This underlines that the regulation of the influx of bone marrow-derived monocyte into the liver is an important event in the onset of liver inflammation and the NAFLD progression (68, 69). For instance, a pattern of upregulated chemokines/chemokine receptors has been reported in NASH patients such as CCL3-5/CCR5 and the chemokines CCL2. The hepatic expression of CD44 and CD62E (E-Selectin), which are also involved in recruitment of leukocyte into inflammation sites, were also strongly upregulated in NASH patients (71, 72). CD44, which interacts with extracellular matrix components (osteopontin, E-selectin, and hyaluronan), regulates the recruitment of macrophages into the liver but also their activation mediated by DAMPs, PAMPs, and saturated fatty acids (72). In human, liver CD44+ cells correlated with NASH, NAS, and liver injury in obese patients (72). The CCL2/CCR2 pair is also a key player in the recruitment of inflammatory monocytes into the injured liver and drives hepatic fibrosis (73, 74). Pharmacological inhibition of CCL2 in murine models of steatohepatitis (MCDD) and chronic hepatic injury (chronic CCl4 treatment) reduced monocyte/macrophage recruitment into the liver and ameliorated hepatic steatosis development (69). CCR2 inhibition by the small molecule inhibitor CCX-872 also decreased the infiltration of CD11b^+^CD11c^+^F4/80^+^ monocytes into the liver and improved glycemic control and liver inflammation, injury and fibrosis in murine models of NAFLD (high fat high fructose diet) (75). Therapeutic treatment with dual antagonist of chemokine receptor CCR2/CCR5, which is under clinical investigation for fibrotic NASH, will be discussed later in the review.

A number of studies using single cell technologies [reviewed in (43)] or specific markers such as C-type lectin CLEC4F, TIM4, and osteopontin (76–78) highlight the complexity of the NAFLD pathogenenis and role of the liver macrophages. Interestingly, it has been recently identified using single-cell RNA sequencing, a common “NAFLD myeloid phenotype” in mice upon Western diet challenge. The liver monocytes, macrophages, and dendritic cells, as well as bone marrow precursors displayed, for example, the downregulation of the inflammatory marker calprotectin (S100a8/S100a9). In addition, the inflammatory capacity of bone marrow monocytes is modified and this phenotype remains stable and independent of the local micro environment and *in vitro* stimulation with cytokine (79). Current efforts are thus focused on dissecting macrophage heterogeneity and potential NAFLD phenotype according to NAFLD severity to allow the development of more specific therapeutic tools to target “detrimental” macrophages and inflammation.

### Innate Lymphoid Cells Are New Participants in NASH

The family of innate lymphoid cells (ILC) represent subsets of innate lymphocytes lacking the receptors of antigen encoded by rearranged genes and expressed on T and B cells. These cells are mainly resident cells in tissue and enriched in the epithelial barrier. ILCs are prompted to respond to various stress signals (pathogens, tumors, and inflammation) and secrete a wide range of cytokines to shape immune responses (80). The ILC family is classified into three groups and five subsets based on cell surface markers, transcriptional factors required for their development and the patterns of producing type 1, type 2, and Th17 associated cytokines: the cytotoxic NK cells and helper-like ILC-1 belonging to group 1 ILC; helper-like ILC-2, the unique group 2 ILC member; helper-like ILC-3 and Lymphocyte Tissue Inducer (LTi) cells belonging to group 3 cells (81). In addition to their role in orchestrating protective immunity, ILC subsets also regulate obesity-associated metabolic diseases and may contribute to NAFLD pathogenesis (24, 82).

Over the last few years, it has been reported that almost all subsets of ILC play an important role in metabolic homeostasis by regulating adipose tissue, liver, and gut functions. Two main studies demonstrated the importance of the IL-33/ILC2 axis in adipose tissue to regulate obesity. Enkephalin and IL13-producing ILC2 promote “beiging” of white adipocytes and increased energy expenditure by regulating eosinophil/alternatively activated macrophage differentiation (83, 84). The IL22 expression by ILC3 subsets was impaired in obese mice. Interestingly, IL22-producing ILC3 or IL22-producing CD4 T cells improved insulin sensitivity, preserved the mucosal barrier of the gut, decreased inflammatory responses, and regulated the lipid metabolism in both adipose tissue and liver (85). The contribution of group 1 ILC in the regulation of adipose tissue inflammation was also established in obese patients and murine models. The NK cells producing IFN-γ were increased in adipose tissue and the depletion of NK cells and/or helper-like ILC1 decreased the number of adipose tissue pro-inflammatory macrophages (86–89). Most recent studies deciphered the phenotypical and functional heterogeneity of adipose tissue group 1 ILC in human and mouse studies and revealed their complexity.

In response to local IL12 and/or IL15 levels, the group 1 ILC subsets produced IFN-γ and TNF-α and regulated the pool of macrophages into the adipose tissue during obesity (90, 91). Interestingly, the increased susceptibility to infection and cancer related to obesity have been associated with the decrease in anti-cytotoxic properties of NK cells. The lipid accumulation in NK cells and PPAR-mediated mTOR inhibition impaired the NK cells functions (92, 93). In NASH patients, the circulating levels of IL15 and CXCL10 increased compared to lean subjects. These inflammatory mediators are known to trigger group 1 ILC activation (71, 94, 95). While circulating NK cells frequency did not change with the grade of NAFLD (NASH versus steatosis), these cells expressed a higher level of the activating receptor NKG2D and were thus be more sensitive to cell death signals (96). Characterization of tissue group 1 ILC according to the hepatic complications still needs to be improved in patients. However, the contribution of group 1 ILC in NAFLD progression has been established. The liver IFN-γ producing NK cells enhanced macrophage polarization towards a pro-inflammatory phenotype with steatohepatitis (MCDD) (44). In addition, the depletion of group 1 ILC exacerbated the NASH-fibrosis transition confirming the anti-fibrogenic role of NK cells/ILC1 (97). Several studies also reported that elevated CXCL10 and IL15 levels in NASH liver contributed to the recruitment and activation of hepatic NK cells/ILC1 (98, 99). The frequency of NK cells in liver could also be regulated by their conversion into the less cytotoxic ILC1-like phenotype during NASH in response to elevated TGF-β (100). The mechanisms regulating the functions of the ILCs and their crosstalk with the other immune cells during NAFLD/NASH deserve more attention in the future to better understand NAFLD pathogenesis.

### Involvement of Neutrophils in NAFLD Pathogenesis

Neutrophils have a well-established role in alcoholic liver diseases (101), and could also be actors contributing in the onset and progression of NASH. Indeed, it has been reported that the targeting of neutrophils (depletion, inhibition of activity, or recruitment) reduced liver inflammation in obesity and steatohepatitis contexts. The depletion of neutrophils via a specific antibody (1A8 targeting Ly6G molecule) improves metabolic parameters and hepatic steatosis and inflammation associated with a reduction of the body weight in HFD mice (102). Neutrophil elastase deficiency decreases the liver steatosis and inflammation in Western diet fed mice (103), while the myeloperoxidase (MPO) deletion ameliorates hepatic inflammation and fibrosis in HFD mice (104). The increased MPO secretion by leukocytes could directly promote hepatocyte injury and hepatic stellate cell activation (45). Neutrophil derived peptides may also contribute to the NAFLD progression. Transgenic mice expressing human neutrophil peptide 1 displayed an exacerbation of hepatic stellate proliferation and fibrosis when fed a choline-deficient, L-amino acid-defined diet (105). Furthermore, neutrophil extracellular traps (NETs), which limit infection by entrapping pathogens, have been linked to chronic sterile inflammation. Van der Windt *et al.* have recently reported that the circulating levels of markers of NETs increase in NASH patients and the liver NET formation occurs at the early stage of the NAFLD (before the influx of monocyte-derived macrophages) in mice. Finally, the inhibition of the NET formation protects mice from hepatic inflammation and NASH-driven HCC (46).

The development and progression of NAFLD are thus multi-factorial and multi-organ. The chronic inflammation is a key player and its indirect or direct targeting as therapeutic approaches will be successively discussed.

## Global Approaches Against NAFLD/NASH

Lifestyle changes are a promising therapeutic approach against NAFLD and would optimize the action of the future pharmacological treatments when they are combined. Indeed, recent reviews summarize the benefits of nutritional management and physical activity on NAFLDs (106, 107). Weight reductions of ≥10% has been associated with the resolution of NASH, in many cases, and the improvement of fibrosis by at least one stage. The modest weight loss (>5%) is also associated with benefits on some items encompassed in the NAFLD activity score (NAS). For example, a 5% reduction in BMI has been associated with 25% reduction in fat in liver according to a Magnetic Resonance Imaging (MRI) measurement (108), with up to complete correction after few weeks under a strictly hypocaloric diet. These global approaches are associated with the improvement of systemic inflammation, adipose tissue inflammation, insulin-sensitivity, and gut functions (eubiosis, integrity, metabolites, and hormones) contributing to the normalization of the insulin-sensitivity and lipid profile. However, the ideal diet (the Mediterranean diet has been proposed as one such diet) and the most effective regular physical activity are yet to be defined by long-term studies. These adapted lifestyle modifications towards a healthy diet and habitual physical activity would also be a therapeutic approach to reduce NAFLD and its cardiovascular and renal complications.

Regarding the impact of the bariatric surgery on NAFLD (109, 110), a recent meta-analysis including 32 cohort studies with 3093 paired liver biopsies reported a resolution of steatosis, inﬂammation, ballooning degeneration and fibrosis in 66, 50, 76, and in 40% of patients, respectively. In line with this, mean NAFLD activity score was also reduced after bariatric surgery. The included studies in this analysis were conducted between 1995 and 2018 with 5 retrospective and 17 prospective cohort studies employing diﬀerent bariatric procedures. The median follow-up duration was of 15 months (3–55 months), with an absolute percentage of BMI reduction of 24.98% after surgery (110). Recent longitudinal studies with paired liver biopsies also reported the beneficial effects of bariatric surgery against NASH in a long term. One study reported that Laparoscopic Roux-en-Y Gastric Bypass surgery resulted in correction hepatic steatosis, inﬂammation, hepatocellular ballooning, and liver injury as evaluated by alanine aminotransferase and liver activated cleaved caspase-3 levels after a median follow-up of 55 months. Interestingly, the hepatocyte apoptosis as evaluated by serum caspase-generated keratin-18 fragment levels already improved one year after the gastric surgery (111). Recently, a study evaluated the impart of bariatric surgery (including diﬀerent procedures) in biopsy-proven NASH patients at 1 and 5 years after the surgery. From the analysis of the sequential liver biopsies, NASH resolution were observed in 84% of cases after 5 years and the reduction of hepatic fibrosis was progressively decreased at 1 year and then 5 years after the bariatric surgery (112). Since the efficacy of bariatric surgery on NASH in patients with BMI ≥35 kg/m^2^ looks promising and efficient, the extension of bariatric surgery indication to patients with a BMI of less than 35 kg/m^2^ is currently being considered. In line with this, the FDA (Food & Drug Administration) has recently approved gastric band indication for obese patients (BMI 30–35 kg/m^2^) with severe type 2 diabetes.

## Pharmacological Targets Against NASH

**Figure 3:**
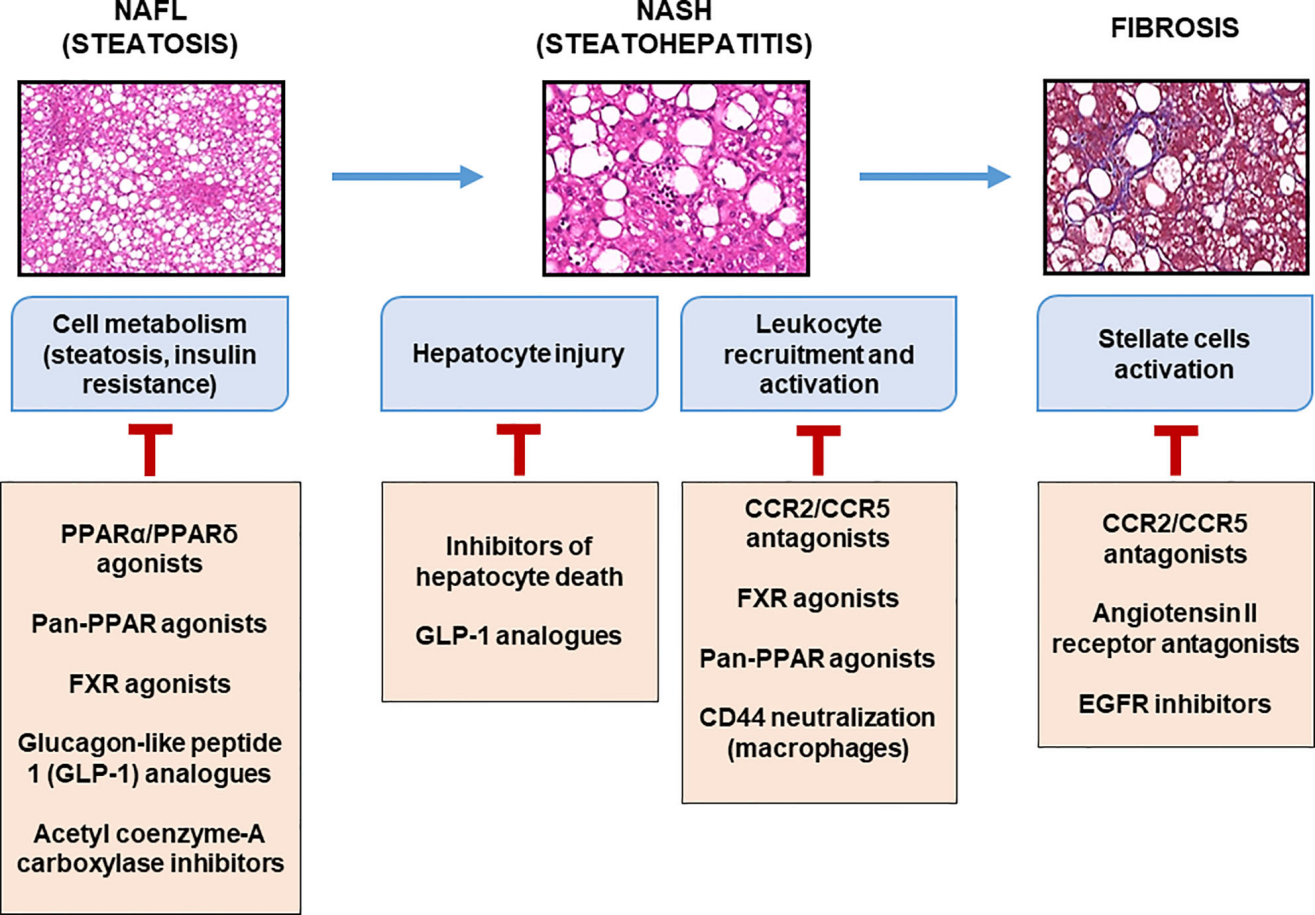
Therapeutic targets at different stages of liver complications. Main molecules targeting metabolic and inflammatory mediators expressed during the progression of liver complications of obesity are listed with some of which are currently in clinical evaluation.

In the near future, pharmacological innovations may be available for patients with fibrotic-NASH. An increasing number of pre-clinical and clinical studies are in progress targeting “metabolism-inflammation-fibrogenesis”. Some compounds target hepatocyte deaths (driver of inflammation; pan-caspase inhibitor, etc.), inflammation and/or fibrosis (CCR2/CCR5 antagonist, galectin-3 inhibitor, etc.), others the metabolism (PPAR pan-agonists, FGF21 agonists, ACC inhibitor, etc.), or the gut-liver axis (FXR agonists, non-tumorigenic analogues of FGF19, etc.) (113). The combination of two or more of these compounds is a rational strategy that is currently under development. The impacts of some of these pharmacology strategies on inflammation are discussed below (**Figure 3**).

### Targeting the Liver Injury (Hepatocyte Death)

As previously described, different types of hepatocyte death have been associated with NAFLD progression and drive hepatic inflammation (40). The targeting of apoptotic caspases such as the pan-caspase inhibitor Emricasan, while effective in preclinical studies (114), did not improve clinical aspects nor NASH features in NASH patients with fibrosis but, to the contrary, could aggravate fibrosis and hepatocyte ballooning (115, 116) (ENCORE-PH and ENCORE-NF trials; Phase II). Likewise, targeting of apoptosis signal-regulating kinase 1 (ASK1) did not prevent fibrosis in NASH patients with severe fibrosis. It has been demonstrated that ASK1, by regulating the sustained activation of JNK, is an important mediator of hepatocyte death and inﬂammation in hepatocytes and macrophages in preclinical and *in vitro* studies. The proof-of-concept study evaluating selonsertib (an ASK1 inhibitor) after 24 weeks also reported an improvement of hepatic ﬁbrosis but not ballooning or inﬂammation (117). However, two placebo-controlled phase III trials in NASH patients with compensated cirrhosis or with bridging fibrosis (STELLAR-4 and -3 trials, respectively) recently reported that Selonsertib did not improve fibrosis as evaluated by noninvasive tests without worsening of NASH after 24 weeks (118). Altogether, these studies could allow us to exclude the ASK1 inhibition strategy for burned out NASH (severe fibrosis). Both of these strategies (inhibition of pro-apoptotic caspases and ASK1) also indicate that prevention of apoptosis may have caused the stressed hepatocytes to enter alternative modes of cell death such as necrosis, necroptosis and pyroptosis (more deleterious by generating more inflammatory mediators). For example, the pan caspase inhibition by Emricasan in pre-treated acute myeloid leukemia cells with an apoptosis enhancer (birinapant) enhances necroptosis at the expense of apoptosis (119). Decrease in caspase 8 activity by Emricasan could explain this shift of cell death by removing the inhibition of necroptosis by caspase 8. New inhibitors targeting necroptosis and pyroptosis are currently being evaluated but particular attention should also be paid to their impact on other modes of cell death which can negate the desired therapeutic benefits.

### Inhibition of Monocyte-Derived Macrophage Recruitment

Limiting the pool of recruited monocyte-derived macrophages is also a promising therapeutic strategy. For example, CD44 neutralization by specific antibody decreases macrophages infiltration into adipose tissue, weight gain, fasting glycaemia, insulin resistance and hepatic steatosis in a dietary mouse model of obesity (120). In addition, CD44 neutralization partially corrects liver injury and inflammation associated with decreased liver neutrophils and macrophages in rodent model of diet-induced steatohepatitis (72). The approaches targeting the CD44 functions or expression in macrophages, for example, could be thus beneficial against NASH. Regarding the CCR2/CCL2 and CCR5/CCL5 systems, an oral dual CCR2/CCR5 antagonist, cenicriviroc (CVC), has been developed and is currently being evaluated in NASH patients. CVC treatment decreased the recruitment of Ly-6C^+^ monocyte-derived macrophages into the liver in mouse models of steatohepatitis (MCDD or Western diet) and ameliorated insulin resistance and liver steatosis. Moreover, CVC treatment improved histological NASH features and liver ﬁbrosis without delaying ﬁbrosis resolution after injury cessation (68). Indeed, subsets of macrophages (Ly6C^low^ restorative macrophages) are also associated with the resolution of fibrosis by secreting anti-inﬂammatory cytokines and collagen degrading factors (33). In addition, prolonged high-dose CVC therapy (14 weeks) in choline deficient, L-amino acid-defined, high-fat diet (CDAHFD) mice, augmented the frequency of intrahepatic anti-inflammatory macrophages without impacting the total intrahepatic macrophage populations and decreased liver fibrosis. The beneficial effect of CVC on fibrosis has been associated with its direct effect on hepatic stellate cells. Indeed, CVC treatment prevented the pro-fibrotic gene signature mediated by transforming growth factor-β in primary mouse hepatic stellate cells (121). In addition to CCR2 inhibition, CCR5 inhibition by CVC could be involved in the prevention the activation, migration and proliferation of hepatic stellate cells (74, 122). CCR5 deficiency reduced hepatic fibrosis mediated by bile duct ligation (122) and CCL5 inhibition displayed similar effects in the carbon tetrachloride rodent model (123). Inhibitor of CCR5 (Maraviror) also arrested cell cycle progression and decreased the accumulation of collagen in the human stellate cell line (124). Among the molecules directly targeting inflammation in clinical trials in phase 2/3 [inhibition of plasma Amine Oxidase Copper-containing 3 (BI 1467335, Boehringer, phase 2) and Galectin 3, a lectin family member (GR-MD-02, Galectin Therapeutics, phase 2)], only CVC (CCR2/CCR5 antagonist, Allergan) is currently under evaluation in a phase III trial in fribrotic NASH patients. In a phase II trial in 289 patients with NASH, CVC therapy for one year was associated with improvement in hepatic fibrosis without worsening of NASH in large part of the patients compared with placebo (125). Results of the phase IIb study of belapectin (Galectin 3 inhibitor) assessed in 162 patients with NASH, portal hypertension and cirrhosis has been recently published. Unfortunately, one year of biweekly infusion of belapectin was not associated with a significant reduction in hepatic venous pressure gradient or fibrosis compared with placebo (126). In a rodent model of NAFLD, inhibitors of galectin 3 prevented hepatic fibrosis, possibly via macrophages (127). Several other compounds targeting the CCR2/CCL2 and/or CCR5/CCL5 systems will likely be evaluated in fribrotic-NASH patients, either alone or combined with other drugs.

### When Metabolism Meets Inflammation

Over the last decade, it has been well established that the metabolism of immune cells drives their immune responses and/or polarizations. The modulation of the activity of peroxisome proliferator-activated receptor (PPAR) family members has been linked to the improvement of insulin sensitivity and reduction of NAFLD in preclinical studies. PPARγ agonists are insulin sensitizers that act mainly in adipose tissues by increasing a pool of insulin-sensitive adipocytes. Since agonists of PPARγ also enhance the anti-inflammatory polarization of macrophages, these agonists also display anti-inflammatory actions in an obesity context (128–130). Several clinical trials have suggested effective PPARγ agonist pioglitazone activity against NASH, but clinical limitations of this drug have been reported relative to the weight gain, the risk of bladder cancer, and potential aggravation of heart failure (131–133).

Regarding recent PPAR-γ agonist, twenty-four-week treatment of type 2 diabetes patients with NAFLD with Lobeglitazone has been associated with a modest weight gain (compare to pioglitazone) and the improvement of glucose homeostasis, lipid proﬁle but also hepatic steatosis. Unfortunately, Its eﬃcacy against NASH still needs to be assessed due to the absence of biopsy-proven NASH in these studies (134–136). PPARα is significantly expressed in liver and regulates metabolism such as bile acid synthesis, ketogenesis, fatty acid uptake, beta oxidation, and triglyceride turnover (137). Importantly, PPARα also displays anti-inflammatory effects by regulating the NF-κB pathway (138). Regarding the last isoform, PPARδ is most highly expressed in muscle but also in adipose tissue and liver. In muscle, the role of PPARδ has been mainly associated with the regulation of mitochondrial metabolism and beta oxidation (139). Regarding its hepatic expression, PPARδ is expressed in hepatocytes but also in hepatic macrophages and stellate cells suggesting its potential contribution in the regulation of liver inflammation and fibrosis (137). Moreover, PPARδ also shifts the Kupffer cells polarization to an anti-inflammatory phenotype (140).

To target both PPARα and PPARδ pathways, dual agonists has been generated such as elafibranor (also known as GFT505). It is important to underline that Elafibranor displays 10 times more affinity for PPARα than PPARδ. In animal models of NASH, elafibranor treatment decreased the numbers of macrophage in the liver (141). In a phase II trial in NASH patients, elafibranor treatment was associated with a greater resolution of NASH compared with placebo without worsening the liver fibrosis (142). However, GENFIT recently reported the intermediate results from the phase III trial (RESOLVE-IT) evaluating elafibranor (120 mg elafibranor once daily) compared to placebo in fibrotic NASH patients (biopsy-proven NAS ≥4 and F2/F3) with a follow-up liver biopsy at week 72. The response rate relative to resolution of NASH with not worsening of fibrosis (primary endpoint) was 19.2% in elafibranor arm (138/717patients) to 14.7% for placebo arm (52/353 patients) without achieving statistical significance (p = 0.0659). Regarding fibrosis improvement of at least one stage, the response rate was 24.5% in elafibranor arm (176/717 patients) and 22.4% in the placebo arm (79/353 patients) (p = 0.4457). In addition, endpoints related to improvement of at least one stage and changes in metabolic parameters (triglycerides, HDL cholesterol, non-HDL cholesterol, LDL cholesterol, HOMA-IR in non-diabetic patients, and HbA1c in diabetic patients) did not achieve statistical significance. While elafibranor did not exhibit a statistically significant effect on NASH resolution, other clinical trials with different co-agonists of PPAR are currently under investigation in NASH patients [PPARα/γ agonism (Saroglitazar, Zydus, phase II) and PPARα/γ/δ agonism (Lanifibranor, Inventiva, phase II), etc.]. From recent Inventiva’s press release regarding the Phase IIb NATIVE clinical trial in NASH patients, the pan-PPAR agonist Lanifibranor meets a statistically significant decrease (p = 0.004) in at least two points in the SAF activity score (combining hepatocellular inflammation and ballooning), compared to baseline, with no worsening of fibrosis at the dose of 1,200 mg/day (49% in Lanifibranor arm versus 27% in the placebo arm) after 24 weeks of treatment. Lanifibranor also meets multiple key secondary endpoints including fibrosis improvement (by at least one stage without NASH worsening), insulin resistance (decreased in insulin, fasting glucose, Hb1Ac), lipid profiles (decreased in insulin, fasting glucose, Hb1Ac and triglycerides and increased in HDL), and liver injury (decreased in ALT, AST, and GGT).

Other drugs indirectly affect inflammation in NAFLD. The agonist of farnesoid X receptor (FXR) such as obeticholic acid improves liver lipid and glucose metabolism and dampens liver inflammation and fibrosis in NAFLD. In addition, FXR agonists decreases the expression of pro-inflammatory cytokines in macrophages and hepatic inflammation in a mouse model of NAFLD (143). FXR agonists could also enhance the anti-inflammatory polarization of the macrophages *in vitro* and *in vivo* (144). In The Lancet, Younossi *et al.* recently reported the intermediates outcomes (after 18-month of treatment) of a phase III study evaluating the safety and efficacy of daily dose of 10 or 25 mg of obeticholic acid in 931 patients (with 58% females) with F2/3 fibrosis (fibrosis evaluated on liver biopsy) (145). In NASH patients, obeticholic acid (at 25 mg) significantly improved liver fibrosis and some items of NASH disease activity. Although these encouraging results of this phase III trial, some questions persist (the long-term clinical benefits of treatment of NASH, metabolic consequences, management of side effects including pruritus and elevated LDL cholesterol in patients with elevated risk of cardiovascular disease, etc.).

## Conclusion

Our understanding of the pathogenesis of NAFLD with global and specific outcomes is in constant progress. The advances in *in vitro* and *in vivo* approaches are also important issues. With their own limitations, these complementary approaches allow to better highlight novel actors and mechanisms involved in the onset and progression of liver complications. Novel animal models and specific cell isolation combined with single-cell RNA sequencing are examples (146). Liver organoids also emerge as alternative system with multiple hepatic cell types which mimic liver structure and diseases (147). For example, liver organoids from human pluripotent stem cells could be used as model of NAFLD liver when stimulated with free-fatty acids (148). Primary liver organoids according to the severity of NASH have also been successfully generated from mice. These different NASH organoids also display the upregulation of TNFα and IL1β at the early stage of NASH, for example (149). Pre-clinical studies and some clinical trials demonstrate promising results but also underline the complex nature of these chronic liver diseases. Combined metabolic improvement with the regulation of specific inflammatory responses are important clues. The impacts on NAFLD of targeting the GLP1 and hepatic thyroid hormone (thyroid hormone receptor-β) pathways are under clinical evaluation. Promising investigations are currently deciphering the pathways that regulate both hepatocyte death (more specifically lytic cell death) and metabolism but also control inflammation (necroptosis). In addition, the development of new strategies to regulate the immune system and gut microbiota interactions are also promising therapeutic strategies.

## Author Contributions

CL, MB, PL, RA, and PG wrote and edited the paper and approved the final submitted draft. All authors contributed to the article and approved the submitted version.

## Funding

This work was supported by grants from INSERM (France), charities [Association Française pour l’Etude du Foie (AFEF) to PG, Société Francophone du Diabète (SFD) to PG, SFD/Roche Pharma to PG]. This work was also funded by the French Government (National Research Agency, ANR): #ANR-18-CE14-0019-02, ANR-19-CE14-0044-01 and through the “Investments for the Future” LABEX SIGNALIFE (#ANR-11-LABX-0028-01) and the UCA^JEDI^ Investments in the Future project (#ANR-15-IDEX-01). MB is supported by the ANR (#ANR-18-CE14-0019-02).

## Conflict of Interest

The authors declare that the research was conducted in the absence of any commercial or financial relationships that could be construed as a potential conflict of interest.
